# Fabrication of single-crystalline plasmonic nanostructures on transparent and flexible amorphous substrates

**DOI:** 10.1038/srep42859

**Published:** 2017-02-20

**Authors:** Tomohiro Mori, Takeshi Mori, Yasuhiro Tanaka, Yoshifumi Suzaki, Kenzo Yamaguchi

**Affiliations:** 1 Department of Advanced Materials Science, Faculty of Engineering, Kagawa University Hayashicho 2217-20, Takamatsu, Kagawa 761-0396, Japan; 2 Industrial Technology Center of Wakayama Prefecture Ogura 60, Wakayama, Wakayama 649-6261, Japan

## Abstract

A new experimental technique is developed for producing a high-performance single-crystalline Ag nanostructure on transparent and flexible amorphous substrates for use in plasmonic sensors and circuit components. This technique is based on the epitaxial growth of Ag on a (001)-oriented single-crystalline NaCl substrate, which is subsequently dissolved in ultrapure water to allow the Ag film to be transferred onto a wide range of different substrates. Focused ion beam milling is then used to create an Ag nanoarray structure consisting of 200 cuboid nanoparticles with a side length of 160 nm and sharp, precise edges. This array exhibits a strong signal and a sharp peak in plasmonic properties and Raman intensity when compared with a polycrystalline Ag nanoarray.

The surface plasmon (SP) oscillation mode is caused by the nanoscale integration offered by coupling the energy of a photon and electron along a metal-dielectric interface. The unique capability of SPs for subwavelength confinement and electric field intensity enhancement has led to them being widely used in optical nanoantennas^1^,^2^,^3^, surface-enhanced Raman scattering (SERS) sensors^4^,^5^, subwavelength waveguides^6^,^7^, plasmonic vortex lens^8^, and colour filters^9^,^10^. The performance of these plasmonic devices, however, is largely determined by the dielectric properties of the metal used for their fabrication and the precision of their structure. Indeed, most metals used for plasmonic devices are subject to large optical losses due to a scattering of conduction electrons by lattice defects and grain boundaries^11^. This damping of the SP influences the imaginary component of dielectric permittivity representing optical losses^12^, i.e., an increase in the imaginary component is accompanied by a decrease in the enhancement of electric field intensity and propagation length offered by the SP^12^,^13^, particularly in polycrystalline metals with many inner defects. In contrast, the smaller imaginary component of the dielectric permittivity of metals (i.e., the smaller SP damping of a single-crystalline metal) is expected to produce lower energy losses. The fabrication of single-crystalline metallic nanostructures has been reported using chemical reduction methods^14^,^15^, but when they are transferred from a liquid solution to a substrate, it is difficult to control their position and areas due to random massing of the nanostructures. It would therefore be preferable to fabricate metallic nanostructures using a physical technique, such as depositing them onto a substrate by evaporation or sputtering deposition, followed by top-down focused ion beam (FIB) milling or electron beam (EB) lithography. Either approach can provide nanoscale resolution, but FIB has the advantage of greatly reducing the number of processes required. In either event, it is advantageous for the metallic nanostructures to be fabricated immediately after milling of the metallic films.

The authors of the present study recently fabricated a single-crystalline Ag nanopillar in a large (111)-oriented grain on a SiO_2_ substrate using FIB^16^, but grain growth limitations restricted its area to <0.5 μm^2^. Nevertheless, this nanopillar produced a stronger signal than the polycrystalline nanopillar in minuscule nanoparticles, thereby confirming that a single-crystalline nanostructure can reduce optical losses. Larger areas of single-crystal, lattice-matched materials such as MgO, LiF, and mica have been used for the hetero-epitaxial growth of noble metals^17^,^18^,^19^. However, none of these methods have succeeded in fabricating a single-crystalline metallic film on only a particular substrate. This has limited their application with plasmonic devices, which are dependent on the properties of the substrate used. For example, although a stable and highly transparent SiO_2_ substrate can provide high transmissivity, its amorphous nature prevents directly obtaining a large single-crystalline area. Similarly, it is very difficult to produce a single-crystalline metallic film on a transparent and flexible polymer. If plasmonic components could be created on transparent, flexible, stretchable, nonplanar, and biocompatible substrates, rather than conventional rigid substrates, potentially providing circuit components new functionalities for next-generation optical devices.

In an attempt to solve optical energy losses due to inner defects or limitations of the substrate, we propose an experimental technique for preparing single-crystalline Ag films on a desired substrate (in this case, a (001)-oriented single-crystal of NaCl). The epitaxial growth of face-centred cubic (FCC) metals has been demonstrated in the past and thoroughly investigated for alkali-halide cubic crystals of NaCl and KCl^20^,^21^. It is well known that Ag films on a (001)-oriented NaCl crystal exhibit a cube-on-cube orientation relationship of Ag(001) // NaCl(001). Based on this, we propose using ultrapure water to remove the NaCl substrate, thereby allowing the Ag film to be transferred onto SiO_2_ or flexible polyethylene terephthalate (PET) film. As this eliminates the need for solvents or mechanical stripping, there should be no damage to the Ag film surface. Patterned nanostructures can then be milled into the single-crystalline Ag film by FIB. The optical properties of the resulting single-crystalline Ag films and nanostructures are herein investigated and compared to a conventional polycrystalline films and nanostructures.

## Results & Discussion

**Figure 1:**
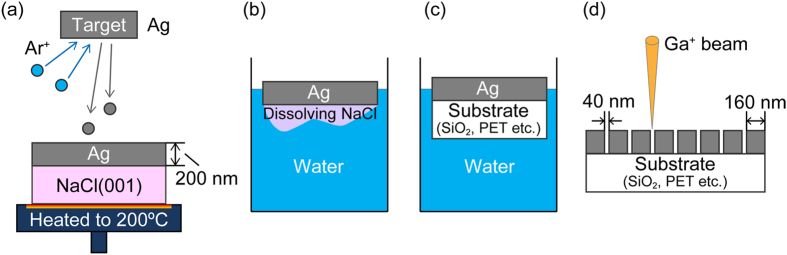
Four-step process for sample preparation. (**a**) epitaxial deposition of Ag film on NaCl(001) substrate by RF magnetron sputtering (substrate temperature: 200 °C, film thickness: 200 nm), (**b**) dissolution of NaCl(001) substrate in ultrapure water, (**c**) adhesion of desired substrate onto Ag film at air-water interface, (**d**) milling of Ag nanoarray into Ag film by focused ion beam.

The crystal growth technique used in this study was based on hetero-epitaxial deposition because it is not possible to directly produce a single-crystalline metallic film on an amorphous substrate. This meant that samples were instead prepared using the four-step process shown in Fig. 1. Here, an Ag film was first deposited onto a cleaved (001)-oriented single-crystalline NaCl substrate via RF magnetron sputtering. The conditions needed for the most highly oriented film were determined (a temperature of 200 °C and film thickness of 200 nm). In the second step, the NaCl substrate was dissolved in ultrapure water at room temperature, as shown Fig. 1(b). Once free, the single-crystalline Ag film floated on the ultrapure water, allowing it to be transferred to a 1-mm-thick SiO_2_ substrate or 100-μm-thick flexible PET film at the air-water interface (Fig. 1(c)). The transferred Ag film was sufficiently vacuum dried to eliminate any interfacial water between the film and substrate. Therefore, the Ag film and substrate adhere uniformly over the whole area by van der Waals’ force, without wrinkles. Moreover, the transferred Ag film on SiO_2_ substrate has demonstrated stability and durability because it did not exfoliate despite immersion in solvents (1-Methyl-2-pyrrolidone, *γ*-Butyrolactone, Toluene, and Acetone). Finally, a nanoarray pattern was milled into the Ag films over an area of 8 × 8 μm^2^ by FIB milling. The resulting nanoarray structure (Fig. 1(d)) consisted of 200 cuboid nanoparticles, each measuring 160 × 160 × 200 nm^3^ (length × width × height) in size, with a gap width (defined as the distance between the sides of two nanoparticles) of 40 nm. For comparative purposes, a polycrystalline Ag film was directly deposited onto an amorphous SiO_2_ substrate using the same deposition and milling condition as for the single-crystalline Ag film.

**Figure 2:**
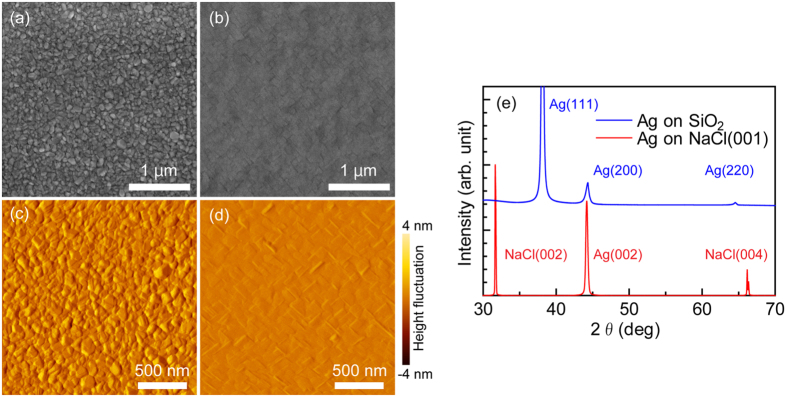
SEM images of (**a**) polycrystalline Ag film on SiO_2_ substrate and (**b**) single-crystalline Ag film on NaCl(001) substrate. AFM images of (**c**) polycrystalline and (**d**) single-crystalline Ag films (colour range shows amplitude of height fluctuations in surface roughness). (**e**) X-ray diffraction patterns of Ag films. The substrate size is 10 × 10 mm^2^.

Figure 2(a) shows the fine-grained Ag thin film that was produced on the SiO_2_ substrate, which is consistent with the “islands” that are typically formed when metallic films are deposited on an amorphous substrate (i.e., Volmer-Weber mode growth). The Ag film produced on the NaCl(001) substrate shown in Fig. 2(b), on other hand, clearly had a smooth surface. The surface roughness and texture of these two Ag films, as characterized by AFM, are shown in Fig. 2(c) and (d). This revealed that the root mean square (RMS) surface roughness of the Ag film on NaCl (0.81 nm) was less than 1/4 that of the Ag film on SiO_2_. This can be explained by the fact that an epitaxial Ag film on a single-crystalline NaCl substrate can form a very flat and smooth surface. The diagonal lines evident in Fig. 2(d) are believed to be caused by a stacking fault of the {111} planes due to inner defects resulting from a lattice mismatch between Ag (a = 4.0862 Å) and NaCl (a = 5.628 Å). As the wide-angle XRD pattern of the Ag film on NaCl (Fig. 2(e)) only contained a Ag(002) peak (in addition to peaks relating to NaCl substrate), it was clearly a single-crystalline film formed in the growth direction. In contrast, the Ag film on SiO_2_ displayed peaks corresponding to Ag(200), Ag(111), and Ag(220), indicating that it was a polycrystalline film. The increase in the low-angle side of the spectrum is caused by a halo pattern from the SiO_2_ substrate.

**Figure 3:**
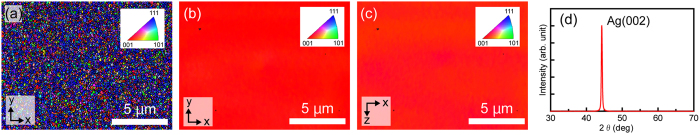
Crystal orientation maps of Ag films on SiO_2_ substrates: z-direction growth of (**a**) polycrystalline and (**b**) single-crystalline Ag films, (**c**) y-direction of single-crystalline Ag film. (**d**) X-ray diffraction pattern of single-crystalline Ag film on SiO_2_ substrate without NaCl peaks.

Figure 3(a–c) present crystal orientation maps obtained using EBSD from the polycrystalline Ag film on SiO_2_, and the single-crystalline Ag film after it was transferred onto SiO_2_ substrates after dissolving its original NaCl substrate. The results in Fig. 3(a) suggest that a polycrystalline Ag film directly deposited onto SiO_2_ has many crystal grains with various orientations, but with a strong (111) texture (as shown in blue). With FCC metals on amorphous substrates, it is thought that both the surface and interface energy are minimized by grains with a (111) texture due to the close-packed plane of the FCC crystal; i.e., the growth of grains with a (111) texture is generally favoured over the growth of grains with another orientation^16^. Figure 3(b) and (c) show the growth of the Ag films in the z- and y- directions, respectively, with Fig. 3(b) revealing that the single-crystalline Ag film had only one crystal orientation in the z-direction. After the single-crystalline Ag film was transferred to a SiO_2_ substrate, only an Ag(002) peak was detected in its XRD spectrum (Fig. 3(d)) once the influence of NaCl was removed, and it had only one crystal orientation in the y-direction shown by Fig. 3(c). Based on this, it is assumed that a single-crystalline Ag film on NaCl(001) exhibits an orientation relationship of Ag(001) // NaCl(001). Although this produces a big misfit, the single-crystalline Ag film grows parallel to the NaCl(001) crystal orientation because it is also influenced by the surface free energy of the substrate, contamination of the substrate and the degree of vacuum^20^,^21^. Furthermore, the crystal growth and the optical dielectric constants of Ag can possibly be affected by the level of water or oxygen contamination in a vacuum. The degree of vacuum and more importantly the deposition rate can influence the level of contaminants in the Ag film.

**Figure 4:**
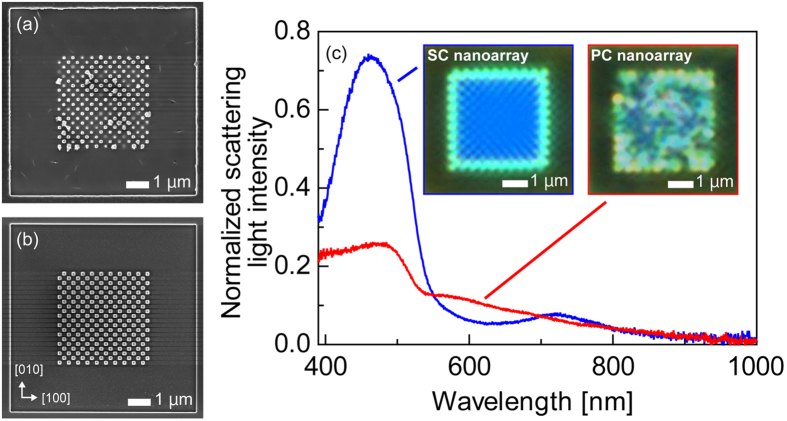
SEM images of (**a**) polycrystalline (PC) and (**b**) single-crystalline (SC) Ag nanoarrays on SiO_2_ substrates. (**c**) Scattered light spectra of PC and SC Ag nanoarrays, which were illuminated with unpolarised light (insets: optical microscope images of each crystal).

The SEM images in Fig. 4(a) and (b) show the polycrystalline and single-crystalline Ag nanoarrays produced on SiO_2_ substrates by FIB. As the polycrystalline Ag nanoarray contained variously shaped nanoparticles, it is assumed that the ion beam etching rate for this film varied depending on the material, crystal orientation, grain size of the rough film, and ion beam incidence angle^22^. This means that a precise nanostructure pattern is not possible under these conditions. In contrast, the single-crystalline Ag nanoarray consisted of cuboid nanoparticles with precise and sharp edges, with this excellent processability the result of the nanoarray pattern being milled along the orthographic (100) and (010) planes. These sharp nanoparticles are expected to be much more conducive to enhancing the electric field at their corners^23^, which means that a single-crystalline film is advantageous to achieving a precise nanostructure pattern. With the use of a single-crystalline film, it is even possible to produce precise circular patterns. The scattered light spectra of the Ag nanoarrays in the 390 to 1000 nm wavelength range shown by Fig. 4(c) reveal that the single-crystalline Ag nanoarray exhibits a strong signal with a sharp peak at around 460 nm relative to the polycrystalline Ag nanoarray. The effect of polarization unfortunately could not be confirmed because of the size of the nanoparticles and large gap width. The outermost nanoparticles of the Ag nanoarray were observed to emit a green light, as these were not surrounded by other nanoparticles. This means that an SP has a resonance wavelength that depends on the refractive index of the surrounding medium^24^.

**Figure 5:**
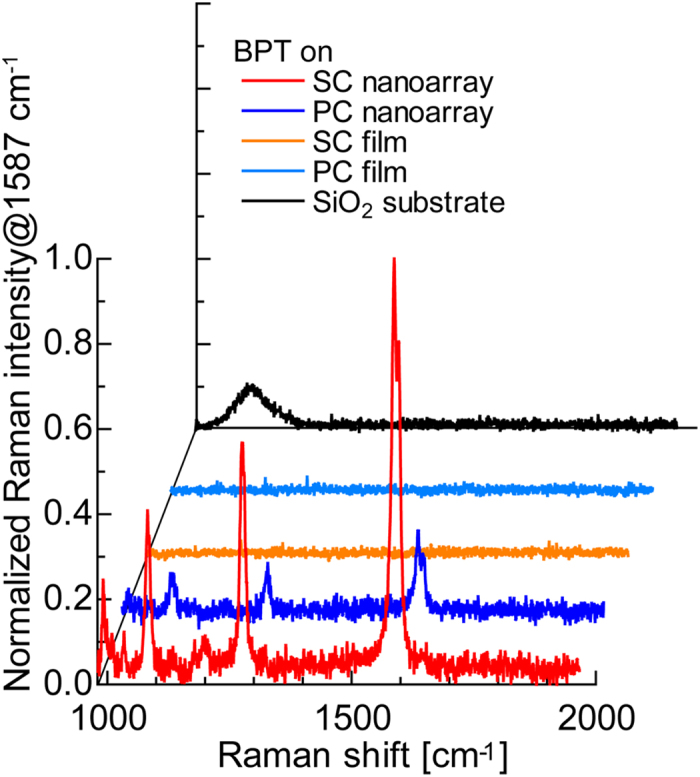
Raman spectra of BPT molecules adsorbed on different substrates recorded at 532 nm with a continuous wave laser (integration time: 60 s).

Figure 5 shows the Raman spectra of 1 mM BPT molecules adsorbed onto different substrates in the 980 to 1860 cm^−1^ wavenumber range. These BPT molecules were irradiated without damage by the incident laser beam (532 nm, ~300 μW/μm^2^), but the resulting Raman signal was so weak that it could not be detected in the case of the single-crystalline Ag film, polycrystalline Ag film, or SiO_2_ substrate. The 1086 cm^−1^ peak for the BPT molecules on SiO_2_ is caused by the *ν*(Si–O) asymmetric stretching vibration mode of the SiO_2_ substrate. The Raman signal was detected coming from the single-crystalline and polycrystalline Ag nanoarrays. The other observed Raman bands were assigned to the ring *ν*(C–H) deformation vibration mode at 992 cm^−1^, 1034 cm^−1^, and 1082 cm^−1^; and the ring *δ*(C = C) stretching vibration mode at 1276, 1587, and 1597 cm^−1 ^25. When both structures are compared, it is apparent that the Raman signal from the BPT molecules on the single-crystalline Ag nanoarray was more than five-times that of the molecules on the polycrystalline Ag nanoarray. This confirmed that SP resonance is efficiently excited by a single-crystalline Ag nanostructure, which can therefore provide an excellent substrate for a SERS sensor.

**Figure 6:**
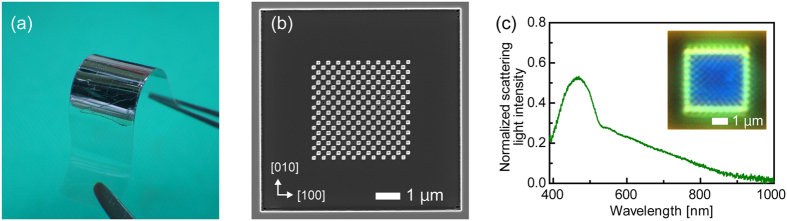
(**a**) Photograph and (**b**) SEM image of single-crystalline Ag nanoarray on flexible 100-μm-thick PET film. (**c**) Scattered light spectrum of Ag nanoarray, which was illuminated with unpolarised light (inset: optical microscope image).

The single-crystalline Ag film was also transferred onto a PET film and milled by FIB, with Fig. 6(a) showing a photograph of the completed process. We confirmed the occurrence of cracks while varying the bend angle (curvature radius) of the film. The Ag film did not present any cracks unless the Ag film was bent to a curvature radius of 1 mm. The resulting single-crystalline Ag film with nanoarray was flexible and durable because of the high ductility of Ag. Moreover, it retained the same precise and sharp edges that were seen on a SiO_2_ substrate in Fig. 6(b), which means that they can be fabricated regardless of the physical properties of the substrate. The scattering spectrum of the single-crystalline Ag nanoarray on PET peaked at around 470 nm, which represents a shift to a longer wavelength compared to using a SiO_2_ substrate. Such a shift has been linked to an increase in the refractive index of the substrate^26^ and has the greatest influence on those nanoparticles positioned on the outside. Consequently, the spectrum had a broad spectral distribution ranging from 500 to 600 nm. After taking a closer look at Fig. 6(b), the nanoarray pattern was processed by FIB just a little too long at a position close to the right from the centre. It is assumed that the strength reduction of scattering light intensity was probably affected by the change in shape of the PET film.

## Conclusions

Through XRD spectra and crystal orientation maps obtained using EBSD, this study has demonstrated that single-crystalline Ag thin films grow epitaxially in a direction parallel to the crystal orientation of a (001)-oriented single-crystalline NaCl substrate. More importantly, this NaCl substrate can be dissolved in ultrapure water, allowing the floating Ag film to be transferred onto transparent and flexible amorphous substrates. Using this approach, a single-crystalline Ag nanoarray with precise and sharp edges was produced on an amorphous substrate and found to exhibit excellent optical properties. As this same experimental technique can be applied to a wide range of substrates, it is expected to aid in the development of various applications, particularly plasmonic sensors and waveguides.

## Methods

### Fabrication of the single-crystalline nanostructures

Samples were prepared using the four-step process shown in Fig. 1. Here, an Ag film was first deposited onto a cleaved (001)-oriented single-crystalline NaCl substrate (10 × 10 mm^2^) via RF magnetron sputtering (ARIOS Corp., Japan) in a chamber evacuated to 1.2 × 10^−4^ Pa. Several sets of samples were produced using substrate temperatures of 20–500 °C, a sputtering power supply of 50 W and an Ar working gas pressure of 0.7 Pa to produce film thicknesses of 100–300 nm at a deposition rate of 5.5 Å/s. From this, the conditions needed for the most highly oriented film were determined (a temperature of 200 °C and film thickness of 200 nm). In the second step, the NaCl substrate was dissolved in ultrapure water (electric conductivity: < 0.06 μS/cm) at room temperature, as shown Fig. 1(b). Once free, the single-crystalline Ag film floated on the ultrapure water, allowing it to be transferred to a 1 mm-thick SiO_2_ substrate (refractive index: 1.521) or 100-μm-thick flexible PET film (refractive index: 1.600) at the air-water interface (Fig. 1(c)). The transferred Ag film was sufficiently vacuum dried at room temperature to eliminate any interfacial water. Therefore, the Ag film and the substrate adhere uniformly over the whole area by van der Waals’ force, without wrinkles. Finally, a nanoarray pattern was milled into the Ag films over an area of 8 × 8 μm^2^ by FIB milling (Quanta3D 200i, FEI Corp., USA) using an accelerating voltage of 30 kV and a beam current of 49 pA. To reduce the influence of the ion beam, the periphery of the Ag nanoarray was processed using an accelerating voltage of 2 kV and a beam current of 49 pA for the final touch. To prevent drifting of the observed image, the Ag nanoarray was monitored during operation in real time. The resulting nanoarray structure (Fig. 1(d)) consisted of 200 cuboid nanoparticles, each measuring 160 × 160 × 200 nm^3^ (length × width × height) in size, with a gap width (defined as the distance between the sides of two nanoparticles) of 40 nm. For comparative purposes, a polycrystalline Ag film was directly deposited onto an amorphous SiO_2_ substrate using the same deposition and milling condition as for the single-crystalline Ag film.

### Measurements

The crystal orientation of the Ag films was evaluated by XRD (SmartLab, Rigaku, Corp., Japan) using a parallel beam method, which has excellent angle resolving power and reduced the intensity of X-rays to which the thin films were exposed. A detailed crystallography of the local area used for milling the nanoarray structure was obtained using an SEM (JSM-7001F, JEOL, Corp., Japan) equipped with an EBSD system by scanning a 15 × 11 μm^2^ area at a step interval of 30 nm. A feature of the EBSD method is that it is able to measure the nanoscale crystallographic texture, which cannot be achieved with XRD. The resulting data was analysed using HKL Channel 5 software (Oxford Instruments Corp., UK). The surface roughness and texture of the Ag films was imaged using an atomic force microscope (AFM, NanoNavi/E-sweep, Hitachi High-Tech Science, Corp., Japan). The light intensity scattered by the Ag nanoarray was measured using a dark-field confocal microscope (BX51TRF, Olympus, Corp., Japan) with an objective lens (100 × , N.A. = 0.90) and then guided to a spectrometer (QE65000, Ocean Optics, Inc., USA) and a CCD camera (INFINITY3-6URC, Lumenera, Corp. Canada). An unpolarised halogen lamp was used as the light source. The signal from the Ag nanoarray was masked by noise (i.e., the characteristics of the light source and detector), and so this was eliminated by measuring the background (without the Ag nanoarray) and a reference (the light source) to obtain the normalized scattering light intensity of the Ag nanoarray alone. To evaluate the performance of the Ag nanoarray as a substrate for a SERS sensor, samples of a SiO_2_ substrate, single-crystalline and polycrystalline film, and single-crystalline and polycrystalline nanoarray were prepared by immersion in a solution of 1 mM biphenyl-4-thiol (BPT) in ethanol for 24 h. A 532-nm continuous-wave laser was attenuated to ~300 μW/μm^2^ using a variable neutral density filter and focused onto the sample using an objective lens (100 × , N.A. = 0.95) to a spot size of ~700 nm with an integration time of 60 s to obtain the Raman spectrum of each sample (NRS-3100, JASCO, Corp., Japan).

## Additional Information

**How to cite this article**: Mori, T. *et al*. Fabrication of single-crystalline plasmonic nanostructures on transparent and flexible amorphous substrates. *Sci. Rep.* **7**, 42859; doi: 10.1038/srep42859 (2017).

**Publisher's note:** Springer Nature remains neutral with regard to jurisdictional claims in published maps and institutional affiliations.

## References

[b1] Agio M. Optical antennas as nanoscale resonators. Nanoscale 4, 692–706 (2012). 22175063 10.1039/c1nr11116g

[b2] Kollmann H. . Toward Plasmonics with Nanometer Precision: Nonlinear Optics of Helium-Ion Milled Gold Nanoantennas. Nano Lett. 14, 4778–4784 (2014). 25051422 10.1021/nl5019589

[b3] Jacassi A., Bozzola A., Zilio P., Tantussi F. & Angelis F. D. 3D coaxial out-of-plane metallic antennas for filtering and multi-spectral imaging in the infrared range. Sci. Rep. 6, 28738(2016). 27345517 10.1038/srep28738 PMC4921826

[b4] Yokota Y., Ueno K. & Misawa H. Essential nanogap effects on surface-enhanced Raman scattering signals from closely spased gold nanoparticles. Chem. Commun. 47, 3505–3507 (2011). 10.1039/c0cc05320a 21318204

[b5] Kondo T., Masuda H. & Nishio K. SERS in Ordered Array of Geometrically Controlled Nanodots Obtained Using Anodic Porous Alumina. J. Phys. Chem. C 117, 2531–2534 (2013).

[b6] Pile D. F. P. . Two-dimensionally localized modes of a nanoscale gap plasmon waveguide. Appl. Phys. Lett. 87, 261114 (2005).

[b7] Choo H. . Nanofocusing in a metal-insulator-metal gap plasmon waveguide with a 3-dimensional linear taper. Nat. Photonics 6, 838–844 (2012).

[b8] Garoli D., Zilio P., Tantussi F. & Angelis F. D. Optical vortex beam generator at nanoscale level. Sci. Rep. 6, 29547(2016). 27404659 10.1038/srep29547 PMC4941733

[b9] Yokogawa S., Burgos S. P. & Atwater H. A. Plasmonic Color Filters for CMOS Image Sensor Applications. Nano Lett. 12, 4349–4354 (2012). 22799751 10.1021/nl302110z

[b10] Yamaguchi K., Fujii M., Okamoto T. & Haraguchi H. Electrically driven plasmon chip: Active plasmon filter. Appl. Phys. Express 7, 012201 (2014).

[b11] West P. R. . A. Searching for better plasmonic materials. Laser Photonics Rev. 4, 795–808 (2010).

[b12] Park J. H. . Single-Crystalline Silver Films for Plasmonics. Adv. Mater. 24, 3988–3992 (2012). 22700389 10.1002/adma.201200812

[b13] Ditlbacher H. . Silver Nanowires as Surface Plasmon Resonators. Phys. Rev. Lett. 95, 1–4 (2005). 10.1103/PhysRevLett.95.257403 16384506

[b14] Guo Z. . Facile synthesis of micrometer-sized gold nanoplates through an aniline-assisted route in ethylene glycol solution. Colloids Surfaces A: Physicochem. Eng. Aspects. 278, 33–38 (2006).

[b15] Zhao H. . Green “planting” nanostructured single crystal silver. Sci. Rep. 3, 1511(2013). 23515002 10.1038/srep01511 PMC3604711

[b16] Mori T., Tanaka Y., Suzaki Y. & Yamaguchi K. Advanced fabrication of single-crystalline silver nanopillar on SiO_2_ substrate. Appl. Phys. Lett. 108, 043102 (2016).

[b17] Bialas H. & Heneka K. Epitaxy of fcc metals on dielectric substrates. Vac. 45, 79–87 (1994).

[b18] Baski A. A. & Fuchs H. Epitaxial growth of silver on mica as studied by AFM and STM. Surf. Sci. 313, 275–288 (1994).

[b19] Fedotov V. A., Uchino T. & Ou J. Y. Low-loss plasmonic metamaterial based on epitaxial gold monocrystal film. Opt. Express 20, 9545–9550 (2012). 22535045 10.1364/OE.20.009545

[b20] Ino S., Watanabe D. & Ogawa S. Epitaxial Growth of Metals on Rocksalt Faces Cleaved in Vacuum. 1. J. Phys. Soc. Jpn. 19, 881–891 (1964).

[b21] Kato T. Epitaxial Growth of Several F.C.C. Metals on KCl, KBr and KI Crystals Cleaved in Vacuum. Jpn. J. Appl. Phys. 7, 1162–1166 (1968).

[b22] Volkert C. A. & Minor A. M. Focused Ion Beam Microscopy and Micromachining. MRS Bull. 32, 389–399 (2007).

[b23] Mori T., Yamaguchi K., Tanaka Y., Suzaki Y. & Haraguchi M. Optical characteristics of rounded silver nanoprisms. Opt. Rev. 23, 260–264 (2016).

[b24] Okamoto T., Yamaguchi I. & Kobayashi T. Local plasmon sensor with gold colloid monolayers deposited upon glass substrates. Opt. Lett. 25, 372–374 (2000). 18059883 10.1364/ol.25.000372

[b25] Kalbacova J. . Chemical stability of plasmon-active silver tips for tip-enhanced Raman spectroscopy. Nanospectroscopy 1, 12–18 (2015).

[b26] Malinsky M. D., Kelly K. L., Schatz G. C. & Van Duyne R. P. Nanosphere Lithography: Effect of Substrate on the Localized Surface Plasmon Resonance Spectrum of Silver Nanoparticles. J. Phys. Chem. B 105, 2343–2350 (2001).

